# Impact of Pulsed Electromagnetic Field Therapy and Aerobic Exercise on Patients Suffering With Hypertension: A Systematic Review

**DOI:** 10.7759/cureus.56414

**Published:** 2024-03-18

**Authors:** Arjavi A Pakhan, Swapna Jawade, Manali A Boob, Kamya J Somaiya

**Affiliations:** 1 Musculoskeletal Physiotherapy, Ravi Nair Physiotherapy College, Datta Meghe Institute of Higher Education and Research, Wardha, IND

**Keywords:** nonpharmacological method, lifestyle modification, aerobic exercise, pulsed electromagnetic field therapy, hypertension

## Abstract

Hypertension is a major preventable risk factor for cardiovascular disease. This review evaluates the effects of pulsed electromagnetic field (PEMF) therapy and aerobic exercise on blood pressure (BP) levels in hypertensive patients. This study incorporated research conducted between 2012 and 2020 that was found through a systematic literature search. The measures used to estimate the improvement in BP include the BP measurements, quality-of-life (QOL) scale, and plasma nitric oxide (NO) level. The examination of the review comprised eight studies. These encompassed studies involving individuals with a systolic BP (SBP) above 140 mmHg and a diastolic BP (DBP) above 90 mmHg; those falling within the age range of 40 to 60 years, including both genders; and patients on antihypertensive medications. The review of selected articles concluded that PEMF therapy and aerobic exercise positively impact BP among individuals with hypertension. Aerobic exercises of moderate intensity including brisk walking, jogging, and cycling type of aerobic exercises help reduce BP and maintain patients' physical fitness. PEMF therapy is a complementary approach that affects the biological system and potential health, positively impacting BP. Results indicate that PEMF therapy can be a nonpharmacological method to manage BP in clinical populations. More thorough research is necessary to understand the best dosage, long-term effects, and comparison between PEMF therapy and aerobic exercise.

## Introduction and background

Hypertension is a widespread medical condition distinguished by elevated pressure in the systemic arteries [1]. It is a significant, globally recognized risk factor for various health complications, including cardiovascular diseases and mortality [2]. Linked to an increased risk of coronary artery disease, hypertension's global impact on morbidity and mortality underscores its importance as a public health concern. Demonstrating successful control over hypertension has been proven to decrease the load of related illnesses and mortality [3]. Despite the effectiveness of available treatments, managing hypertension remains a challenge due to awareness, treatment, and control issues.

Research suggests that many individuals with hypertension are either uninformed about their condition or do not receive appropriate management [4]. This points to improved awareness campaigns, early detection, and proper treatment strategies to tackle this health issue effectively. Hypertension is a disease that impacts older individuals, especially isolated systolic hypertension [5]. Hypertension is commonly diagnosed when the systolic blood pressure (SBP) is 140 mmHg or more or the diastolic BP (DBP) is 90 mmHg or more. It is associated with cardiovascular illness because of endothelial tissue damage, which leads to atherosclerosis. Adiposity, loss of viscoelastic properties in conduct arteries with aging, and stiffness of the vasculature are all variables contributing to hypertension in older persons [6]. In India, the overall hypertension incidence was 29.8% (95% CI: 26.7-33.0) [7]. Pulsed electromagnetic field (PEMF) therapy enhances the production and release of nitric oxide (NO), a key vasodilator, from endothelial cells. Increased NO levels lead to the relaxation of blood vessels, resulting in decreased peripheral vascular resistance and subsequently lowering BP. Secondly, PEMF modulates calcium-calmodulin (Ca2+-CaM) signaling pathways, which regulate smooth muscle contraction in the blood vessels. By influencing these pathways, PEMF promotes vasodilation, further contributing to BP reduction. Additionally, PEMF has been shown to upregulate the expression of vasodilatory peptides such as calcitonin gene-related peptide (CGRP), which enhances vascular relaxation. Furthermore, PEMF therapy may mitigate oxidative stress and inflammation; both of which contribute to endothelial dysfunction and hypertension [8]. Aerobic exercise has been shown to significantly reduce both SBP and DBP in hypertensive patients. Aerobic exercise improves vascular endothelial function, reduces resting heart rate, increases vasculogenesis, and enhances tolerance for ischemia and reperfusion injury. Additionally, it alters the balance between vasodilatation- and vasoconstriction-related cytokines such as NO, prostacyclin, and thromboxane. Psychosocial stress reduction and improved insulin sensitivity are also implicated in its antihypertensive effects. Overall, aerobic exercise serves as a valuable nonpharmacological intervention for managing hypertension [9]. The objective of this study is to assess and contrast the effects of PEMF therapy and aerobic exercise on the BP of individuals with hypertension and to investigate the potential advantages of these methods in terms of reducing BP, enhancing cardiovascular health, and understanding their potential as nonpharmacological options for managing hypertension [10].

Pathophysiology of hypertension

Hypertension is a complex physiological condition involving intricate interplays of various factors that disrupt the normal regulation of BP. Key elements involve heightened systemic vascular resistance, renin-angiotensin-aldosterone system disruption, and vascular endothelial function changes [11]. The renin-angiotensin-aldosterone system holds crucial significance. Renin release triggers the process where angiotensinogen changes into angiotensin I, and then angiotensin-converting enzyme (ACE) further converts it into angiotensin II. The vasoconstrictive impacts of angiotensin II lead to an increase in BP [12].

Furthermore, the adrenal cortex releasing aldosterone encourages sodium and water retention, contributing to increased intravascular volume and pressure. Vascular endothelial dysfunction occurs, characterized by reduced NO bioavailability. NO typically mediates vasodilatation and counteracts vasoconstriction. Its deficiency disrupts this balance, promoting increased vascular tone [13]. Insulin resistance and inflammation also contribute. Insulin resistance impairs vasodilatation and promotes sodium retention, while chronic inflammation damages endothelial cells and initiates atherosclerosis, further compromising vascular function. Genetic predisposition is evident through studies highlighting familial clustering of hypertension. Various genes associated with salt handling, sympathetic nervous system regulation, and vascular function contribute to BP regulation. The intricate interplay of these factors ultimately leads to persistently elevated BP and the potential for detrimental target organ damage, emphasizing the complexity of hypertension's pathophysiology [14].

Clinical manifestation of hypertension

Hypertension is frequently asymptomatic in its early stages, earning it the title "silent killer." However, several clinical manifestations and symptoms may emerge as the condition progresses, indicating the need for diagnosis and management [15]. These include headaches, which though common among hypertensive individuals, are not consistent and can vary in intensity and frequency. Dizziness and vertigo can also result from the impact of elevated BP on blood vessel stability [16]. Moreover, hypertension may affect the delicate blood vessels in the eyes, leading to blurred vision or retinal changes. Uncontrolled hypertension can strain the heart, potentially causing angina or discomfort in the chest arising from diminished blood supply to the heart muscle [17]. Fluid retention from hypertension can lead to pulmonary congestion and subsequent shortness of breath, especially during physical exertion. The increased workload on the heart might lead to fatigue and reduced exercise tolerance [18]. Palpitations and irregular heartbeat can be observed due to elevated BP. Though not a consistent symptom, some individuals might experience nosebleeds due to increased BP. It is essential to highlight that these signs may suggest other medical disorders. Proper diagnosis involves accurate BP measurement and assessment of overall cardiovascular health [19].

Diagnostic investigations

The diagnostic evaluation of hypertension, commonly known as high BP, involves a comprehensive approach to confirm the condition, assess its severity, identify potential underlying causes, and evaluate the impact on various organ systems. This process typically includes a combination of medical history, physical examination, and a range of diagnostic tests [20]. First and foremost, a thorough medical history is obtained from the patient. This includes inquiries about their lifestyle, family history of hypertension, past medical conditions, and any medications they may be taking. Understanding these factors can provide valuable insights into the patient's overall health and risk factors [21].

Accurate measurement of BP is a fundamental step in diagnosing hypertension. Using a sphygmomanometer, healthcare providers measure both SBP (the top number) and DBP (the bottom number). Hypertension is typically diagnosed when these measurements consistently show SBP at or above 130 mmHg and DBP at or above 80 mmHg, confirmed on multiple occasions over several weeks [22]. Following BP measurement, a comprehensive physical examination is conducted. This examination includes assessing various aspects of the patient's health, such as examining the eyes for retinal changes, listening to the heart and lungs, and checking for signs of kidney disease. These examinations help identify any potential organ damage associated with hypertension [23]. Laboratory tests play a crucial role in evaluating hypertension. Blood tests are conducted to assess conditions that may contribute to or be affected by hypertension. These tests may include assessing blood glucose levels to check for diabetes, lipid profiles to evaluate cholesterol levels, renal function tests to assess kidney function, and electrolyte measurements to detect imbalances. Additionally, urinalysis is often performed to identify signs of kidney damage [24]. In some cases, diagnostic imaging studies are required. Echocardiography and renal ultrasound may be conducted to assess the structure and function of the heart and kidneys, respectively. These tests can provide valuable information about potential complications associated with hypertension [25]. Ambulatory BP monitoring (ABPM) is a specialized test in which the patient wears a portable BP monitor for 24 hours. This allows for a comprehensive assessment of BP throughout the day and night, providing a more accurate picture of the patient's BP patterns [26]. Home BP monitoring may also be recommended. Patients can monitor their BP at home to track trends and assess the effectiveness of prescribed treatments [27]. In specific cases, particularly when hypertension is severe or presents atypical features, further evaluation for secondary hypertension may be necessary [28]. This may involve additional tests to investigate potential underlying causes, such as hormonal disorders, including aldosterone levels, and imaging of the adrenal glands [29]. Finally, risk assessment is an integral part of the diagnostic process. Healthcare providers evaluate the patient's cardiovascular risk factors, including smoking status, obesity, diet, and physical activity, to determine the overall risk profile [30].

## Review

Study selection

**Figure 1:**
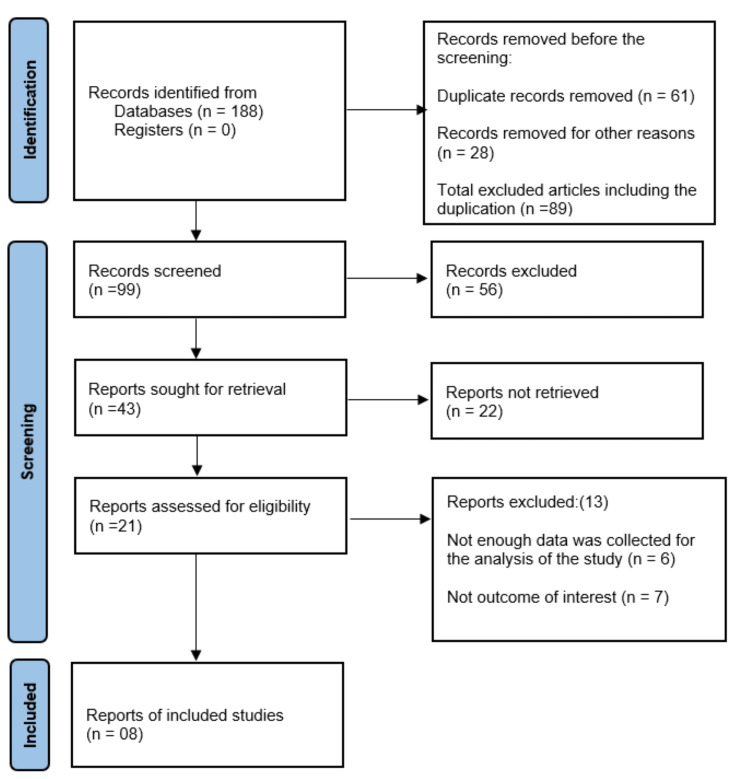
PRISMA chart PRISMA: Preferred Reporting Items for Systematic Reviews and Meta-Analyses

The literature search was conducted using scholarly databases including PubMed, Google Scholar, and ScienceDirect. The keywords used were hypertension, pulsed electromagnetic field therapy, aerobic exercise, and rehabilitation. The search was limited to English language, full-text original research articles. Numerous papers were found, including editorials, reviews, open-access articles, and abstracts. Data were extracted under the headings: author (year), sample size, population, outcome measures, and results were identified. The titles and abstracts were screened for relevance. All of the literature showed improvements in hypertension following either PEMF therapy or aerobic exercise. Out of 188 articles found initially, eight met the eligibility criteria for full-text review. This review adhered to the Preferred Reporting Items for Systematic Reviews and Meta-Analyses (PRISMA) guidelines. Figure 1 shows the database search and the extraction of data. Table 1 shows the summary of all the articles that are reviewed for PEMF therapy and aerobic exercise.and

**Table 1:** Summary of all the articles that are reviewed for PEMF therapy and aerobic exercise BP: blood pressure; SBP: systolic blood pressure; DBP: diastolic blood pressure; FMD: flow-mediated dilatation; PEMF: pulsed electromagnetic field; NO: nitric oxide; HR: heart rate; MD: mean difference; ABP: ambulatory blood pressure; ASI: addiction severity index; AIT: aerobic interval training; TPR: total peripheral resistance; VO2max: maximal oxygen uptake; MIT: moderate intensity continuous training

Sr. no.	Authors and year of publication	Study type	Study sample	Intervention	Results	Conclusion	Interpretation
1	Stewart et al. [8]	Randomized control trial. Outcomes measures: BP measurement, flow-mediated dilatation (FMD)	The sample size of 30 was determined	Group A: PEMF group Group B: control group	The PEMF group showed notable enhancements in FMD & FMD NOR (normalized to hyperemia) with significant improvement in SBP, DBP & MAP (p = 0.05). The control group didn't exhibit the same advancements, with p-values of 0.05 and.04 for FMD and FMD NOR and p-values of 0.04, 0.04, and 0.03 for SBP, DBP, and MAP, respectively	Twelve weeks of PEMF therapy is a successful intervention for improvement in BP and FMD	PEMF treatment helped hypertensive patients lower their BP and enhance endothelial vascular function
2	Kim et al. [31]	Randomized control trial. Outcome measures: BP measurement and plasma NO level	A sample size consists of 44 participants	Group A underwent PEMF therapy; Group B underwent SHAM therapy	Following therapy with PEMF, a notable elevation in NO levels was observed (p = 0.04). Conversely, the SHAM group didn't show a similar effect (p = 0.37)	Incorporating PEMF therapy might augment NO availability in the bloodstream, enhancing BP regulation at rest and during physical activity. Nonetheless, this favorable impact is particularly notable among individuals diagnosed with hypertension	Circulating plasma NO levels were raised after PEMF treatment. When the baseline BP was higher, the effects of PEMF treatment were more pronounced. Following PEMF treatment, circulating plasma NO levels improved
3	He et al. [32]	Pilot study Outcome measures: BP measurement and HR, VO2max, and body fat rate	46 participants	Group A: control group Group B: treatment group	After 12 weeks of brisk walking, the treatment group had significant improvements in BP, HR, physical activity level, cardiovascular fitness, energy expenditure, body composition and sedentary time. Specifically, SBP dropped 8-23 mmHg; HR dropped 3-11 beats per minute; steps rose 6000/day; VO2max rose 2.4 ml/kg/m; moderate activity gained 40 min/day	The 12-week brisk-walking program significantly reduced SBP and HR during resting and exercise, increased physical activity levels, improved cardiovascular fitness, and reduced body fat and sedentary time in elderly patients with essential hypertension	The study indicates that a 12-week brisk-walking program reduces BP, improves cardiovascular health, and enhances physical activity in elderly individuals with essential hypertension, potentially reducing the risk of acute cardiovascular incidents
4	Nascimento et al. [33]	Randomized controlled trial Outcome measures: BP measurement	48 individuals	The participants were randomly allocated into four groups for the short-term interventions (aerobic exercise sessions and control session) on separate days. After these short-term sessions, the participants were randomly allocated into four groups for the 8-week follow-up period (mild-, moderate-, and high-intensity aerobic exercise, and a control group)	The study, involving 48 patients with resistant hypertension, revealed significant reductions in BP levels postexercise across different intensities. This was accompanied by improvements in autonomic modulation and vasodilatory response, highlighting the potential of tailored aerobic exercise regimens as adjunct therapy for managing resistant hypertension	The study underscores the efficacy of customized aerobic exercise interventions in reducing BP levels and enhancing autonomic modulation among patients with resistant hypertension	The findings suggest that tailored aerobic exercise programs could serve as an effective adjunct therapy for controlling BP and improving autonomic function in individuals with resistant hypertension
5	Maruf et al. [34]	Randomized controlled trial outcome measures: BP measurement	63 samples	Group A: control group Group B: exercise group	Significant reductions in both SBP and DBP were observed during the intervention in both the exercise and control groups, with no significant difference between groups post-12-week intervention. BP control rates were comparable between the exercise (56.7%) and control (35.5%) groups, as were the number of antihypertensive drugs required (p = 0.075 and p = 0.511, respectively)	Aerobic exercise combined with antihypertensive drugs showed promising potential in enhancing BP control among individuals with hypertension. Top of Form	Aerobic exercise combined with antihypertensive drugs showed potential in effectively managing BP in individuals with hypertension, suggesting a promising adjunctive therapy for improved hypertension control
6	Rikk et.al. [35]	Randomized study	54 older men and women were included	GROUP A: PEMF therapy GROUP B: SHAM therapy	The SBP and pulse pressure of the PEMF group exhibited a significant decrease with a p-value <0.05. In contrast, the sham group showed a considerable reduction in pulse pressure, although the ASI displayed a tendency toward significance (p = 0.06)	Reductions were observed in systolic and pulse BP, while no significant variations were noted in DBP or the index of arterial stiffness	Improvements in peripheral resistance or circulation may be related to the PEMF therapy; these findings are due interaction of two aspects: The start and progression of hypertension lead to abnormalities in the circulatory system's function and hemodynamic behavior as well as other significant organ structures, as age and many bodily processes change
7	Dimeo et al. [36]	Randomized controlled trial outcome measures: BP measurement, arterial compliance, cardiac index, and physical performance	50 participants were enrolled on the study	Group A: exercise group Group B: control group	The exercise program significantly reduced daytime systolic and diastolic ABPs by 5.9 ± 11.6 and 3.3 ± 6.5 mmHg, respectively, with significant reductions also observed in 24-hour ABPs. Office BP showed numerical reductions without significance. Physical performance increased significantly, evidenced by higher maximal oxygen uptake and maximal workload levels. However, there was no significant correlation between improvements in physical performance and reductions in systolic daytime ABP	The study demonstrates that regular aerobic exercise can effectively reduce daytime ABP in individuals with resistant hypertension, offering a promising adjunctive therapy alongside medical treatments. While exercise improves physical performance, it doesn't significantly impact arterial compliance or cardiac index	The study underscores the efficacy of aerobic exercise in reducing daytime ABP among individuals with resistant hypertension, despite the challenges posed by multiple antihypertensive medications. While exercise enhances physical performance, it does not appear to affect arterial compliance or cardiac index, suggesting its distinct cardiovascular benefits in this population .
8	Molmen-Hansen et al. [37]	Randomized controlled trial outcome measures: ambulatory 24-hour BP	88 patients	88 participants were randomized into AIT, isocaloric MIT, and control groups	24-ABP decreased significantly with AIT by 12 mmHg (systolic) and 8 mmHg (diastolic) compared to 4.5 mmHg (systolic) and 3.5 mmHg (diastolic) with MIT. AIT led to a 15% improvement in VO2max and unique enhancements in TPR reduction and FMD	The research suggests that the effectiveness of exercise in reducing BP among individuals with essential hypertension depends on its intensity. Aerobic interval training emerges as a potent approach for lowering BP and enhancing various cardiovascular risk factors	This study underscores the impact of exercise intensity on hypertension management, with aerobic interval training proving effective in lowering BP and enhancing cardiovascular health markers

Discussion

This study emphasizes the positive effects of two essential interventions: PEMF therapy and aerobic exercise for individuals dealing with hypertension. This thorough review highlights the potential of both approaches to significantly contribute to managing BP and promoting overall cardiovascular health, which fundamentally transforms the panorama of nonpharmacological choices.

PEMF therapy has become a potential treatment for several illnesses, including hypertension. The examined studies consistently provide compelling evidence regarding the effectiveness of PEMF therapy in regulating BP [38]. Research by Stewart et al. [8] and Kim et al. [31] emphasizes PEMF therapy's ability to enhance endothelial function and increase NO levels. Elevated NO levels are directly associated with better BP control, particularly among hypertensive individuals. This substantiates PEMF therapy's role as a nonpharmacological approach that encourages vasodilatation and facilitates nonpharmacological reduction without invasive methods. Aerobic exercise, particularly of moderate intensity, has shown substantial promise in decreasing BP levels for individuals with hypertension, another compelling feature of this issue. Studies led by Cao et al. [39], Punia et al. [40], and Cornelissen et al. [41] highlight the evident benefit of aerobic exercise in lowering SBP and DBP measurements.

Additionally, the study by Rikk et al. [35] reveals improvements in systolic and pulse BP measures in older people, highlighting the benefits of aerobic exercise. This finding emphasizes the importance of incorporating structured aerobic exercise, especially for elderly individuals with hypertension. The potential for interaction between PEMF therapy's impact on NO levels and aerobic exercise's capacity to adjust BP is quite intriguing. The need for thoroughly exploring optimal dosages, long-term viability, and potential combinations of PEMF therapy and various exercise approaches is evident. A more thorough investigation is necessary due to the small number of research, the short period, and the variety of PEMF treatment and aerobic exercise techniques used in the studies. The potential of the best doses, long-lasting benefits, and conceivable interactions between PEMF treatment, aerobic exercise, and more enormous lifestyle changes require extensive exploration. 

## Conclusions

This review article highlights the significance of assessing the impact of therapeutic interventions such as PEMF therapy and aerobic exercise on individuals with hypertension. The analyzed studies underscore the potential benefits of these approaches in improving BP regulation and cardiovascular health. Integrating these interventions into strategies for managing hypertension holds the potential to enhance cardiovascular well-being and elevate the quality of life (QOL) for individuals with this condition. Further research is necessary to determine the sustained effectiveness, optimal application methods, and comparative efficacy of these interventions. Healthcare professionals and researchers should view the findings of this review as a basis for guiding future interventions and enhancing the management of hypertension.
